# Development and internal validation of an interpretable mammography-based model for malignancy risk stratification in BI-RADS 4 breast lesions

**DOI:** 10.3389/fonc.2026.1900262

**Published:** 2026-10-26

**Authors:** Yanheng Zhang, Nan Ding, Tingting Jiao, Bin Wang

**Affiliations:** Linfen People’s Hospital, Linfen, China

**Keywords:** BI-RADS 4, breast cancer, calibration, diagnostic prediction model, internal validation, LASSO, mammography, risk stratification

## Abstract

**Background:**

BI-RADS category 4 breast lesions are suspicious and generally undergo tissue diagnosis, but malignancy risk remains heterogeneous across BI-RADS 4A, 4B, and 4C. We developed and internally validated an interpretable mammography-based model for risk stratification within this diagnostic category.

**Methods:**

This single-center retrospective diagnostic prediction study included 441 patients, each contributing one index BI-RADS 4 mammographic lesion. Outcomes were verified histopathologically in 397 lesions and by longitudinal imaging stability in 44. Three primary models and a sensitivity model excluding BI-RADS were evaluated. Internal validation used 1,000 paired patient-level bootstrap resamples in which imputation, encoding, standardization, penalty selection, LASSO selection, and coefficient estimation were repeated.

**Results:**

Of 441 lesions, 165 were malignant. Malignancy rates were 15.7% (34/216), 46.4% (70/151), and 82.4% (61/74) in BI-RADS 4A, 4B, and 4C, respectively. The final model retained BI-RADS 4B, BI-RADS 4C, architectural distortion, circularity-deficit irregularity score, contour serration score, and normalized GLCM contrast. Optimism-corrected AUCs were 0.770 (95% CI, 0.729-0.815) for BI-RADS only, 0.774 (0.733-0.814) for clinical plus BI-RADS, and 0.841 (0.809-0.877) for the full mammography-based model. Clinical variables did not improve validated performance. The full model improved corrected AUC versus BI-RADS only by 0.072 (0.028-0.108) and versus clinical plus BI-RADS by 0.067 (0.029-0.103). Its corrected calibration intercept was 0.002 (-0.209 to 0.208) and slope was 1.445 (1.140-1.753). Within-category AUCs were 0.785, 0.721, and 0.758 for BI-RADS 4A, 4B, and 4C; the pathology-only corrected AUC was 0.835 (0.799-0.871).

**Conclusion:**

Structured and quantitative mammographic features improved internally validated discrimination and within-category risk stratification beyond BI-RADS subcategory alone. Because predicted risks were under-dispersed and validation was limited to one center, the model should be used only as a research-stage risk-stratification and prioritization tool, not to replace biopsy. External validation and recalibration are required before clinical implementation.

## 1 Introduction

Breast cancer remains one of the most common malignancies in women worldwide, with global estimates from 2022 reporting approximately 2.3 million new female breast cancer cases (1). In China, recent national estimates reported approximately 357,200 new female breast cancer cases and 75,000 deaths in 2022, underscoring the need for accurate diagnostic assessment (2). Mammography remains central to breast cancer detection and diagnostic work-up, but interpretation depends on lesion morphology and background breast parenchyma; mammographic density can influence both cancer risk and detection performance (3, 4).

The American College of Radiology Breast Imaging Reporting and Data System (BI-RADS) was introduced to standardize breast imaging terminology, report organization, assessment categories, and management recommendations (5). The BI-RADS lexicon provides a common language for describing mammographic findings, including mass shape, margins, density, calcifications, architectural distortion, and asymmetry (6). Such standardization is clinically important because mammographic descriptors carry different positive predictive values for malignancy; prior studies have reported higher malignancy probabilities for irregular shape, spiculated or indistinct margins, and suspicious calcification patterns (7–10).

BI-RADS category 4 remains a challenging diagnostic category. It denotes a suspicious abnormality for which tissue diagnosis is generally recommended, yet the expected malignancy probability spans a wide interval. Subdivision into BI-RADS 4A, 4B, and 4C was intended to communicate low, moderate, and high suspicion, but the subcategories still overlap in routine practice (5). Reported positive predictive values for BI-RADS 4 subcategories vary across modalities, institutions, and patient selection mechanisms, reflecting the subjective and setting-dependent nature of risk assignment (11–13).

Several recent studies have attempted to refine risk stratification within BI-RADS 4 lesions. Ultrasound-based nomograms and multimodal models incorporating elastography or automated breast ultrasound have shown encouraging discrimination, but their predictors and workflows are often modality-specific (14–16). Mammography-based deep learning models have also been explored for BI-RADS 4 lesions with the aim of reducing unnecessary biopsies while maintaining diagnostic safety (17). These approaches are promising, but many depend on raw image data, proprietary preprocessing, or complex model architectures that may be difficult to audit in routine reporting environments.

Structured mammographic reports already contain a large amount of clinically interpretable information. A model based on these descriptors, supplemented by transparent quantitative features, may provide a practical middle ground between simple BI-RADS subcategorization and less interpretable image-based artificial intelligence. Interpretable models are also aligned with current reporting guidance for clinical prediction models, which emphasizes clear predictor definitions, transparent modeling, internal validation, calibration, and clinical utility assessment (18, 19).

Therefore, this study aimed to develop and internally validate an interpretable mammography-based model for malignancy risk stratification in BI-RADS 4 breast lesions. We compared BI-RADS alone, clinical variables plus BI-RADS, a full penalized model using clinical and mammographic candidates, and a sensitivity model excluding BI-RADS subcategory.

## 2 Materials and methods

### 2.1 Study design and participants

This retrospective diagnostic prediction study was conducted at Linfen People’s Hospital, a tertiary hospital in China. The protocol was approved by the Ethics Committee of Linfen People’s Hospital (approval number: lf02511253). The requirement for written informed consent was waived by the ethics committee because the study used retrospectively collected and de-identified clinical and imaging data.

Mammographic examinations with structured reports were screened from the institutional database. The verified screening cohort comprised 728 patients. After sequential exclusion of 198 patients with BI-RADS category 3 or 5 findings, 18 with prior breast cancer treatment, 46 without an acceptable reference standard, and 25 with incomplete primary predictor data, 441 patients with BI-RADS 4A, 4B, or 4C lesions were included. Each patient contributed one index lesion, and the patient was the independent sampling unit.

Repeated examinations of the same lesion were excluded, and the first eligible diagnostic mammographic examination was retained. When more than one suspicious lesion was present, the index lesion was selected using an outcome-independent hierarchy: the highest BI-RADS subcategory; if tied, the lesion first scheduled for tissue sampling; and if still tied, the lesion with the larger maximum diameter. Pathology and follow-up outcomes were not used to select the index lesion.

### 2.2 Reference standard and outcome

The primary outcome was lesion malignancy, coded as malignant or benign. Surgical histopathology was used as the final reference standard when available and superseded any preceding percutaneous biopsy result. In the absence of surgical pathology, a radiologic-pathologic concordant core-needle biopsy or vacuum-assisted biopsy result was accepted. Core-needle biopsy used a 14-gauge needle with four to six tissue cores, whereas vacuum-assisted biopsy used a 9-gauge or 11-gauge device. Outcome-derived information was not used as a predictor.

Of the 441 lesions, 397 were histopathologically verified and 44 benign lesions were verified by longitudinal imaging stability through December 31, 2025. The follow-up-verified group comprised 34 BI-RADS 4A, nine BI-RADS 4B, and one BI-RADS 4C lesion. Median follow-up was 34.5 months (interquartile range, 29.0-45.3; range, 24.0-60.0); 14 lesions had two follow-up examinations, 26 had three, and four had four. Follow-up consisted of mammography, supplemented by targeted ultrasonography when clinically indicated.

Imaging stability required no BI-RADS upgrade; no new spiculation, margin indistinctness, architectural distortion, or suspicious density change; no progression in calcification number, extent, morphology, or distribution; and no diagnosis of ipsilateral breast cancer at the same site before the cutoff. For a mass or asymmetry, interval growth required both a relative increase in maximum diameter of more than 20% and an absolute increase of at least 2 mm.

### 2.3 Candidate predictors

Candidate predictors were specified before model fitting on the basis of availability at the index mammographic examination, clinical plausibility, the BI-RADS mammography lexicon, and prior literature, without univariable significance screening. Clinical candidates were age, menopausal status, first-degree family history of breast cancer, body mass index, prior benign breast biopsy, diabetes, hypertension, and palpable mass. Mammographic candidates were BI-RADS 4 subcategory, breast density, lesion type, lesion size, mass shape, mass margin, relative density, calcification presence, calcification number category, calcification cluster extent, architectural distortion, asymmetry, skin or nipple retraction, suspicious axillary lymph nodes, circularity-deficit irregularity score, contour serration score, GLCM texture entropy, and normalized GLCM contrast.

The numerical BI-RADS score and binary dense-breast indicator were excluded because they duplicated BI-RADS subcategory and four-level breast density, respectively. Lesion side and quadrant were retained for cohort description but were not modeled. Mammographic view configuration was excluded because additional diagnostic views could encode work-up intensity. Calcification morphology and distribution were supplementary descriptors because they were conditionally applicable and had greater unavailable information. Not applicable categories were retained as structured categories rather than treated as missing.

### 2.4 Quantitative mammographic feature extraction

For-presentation DICOM mammograms were retrieved from the institutional picture archiving and communication system. Processing accounted for MONOCHROME1 photometric interpretation, RescaleSlope, RescaleIntercept, and PixelSpacing metadata. Image annotations and non-breast regions were removed. Images were resampled to 0.10 x 0.10 mm. Within the lesion-local region, intensities were truncated at the 0.5th and 99.5th percentiles, rescaled to 0-1, and discretized into 64 gray levels.

Lesions were manually segmented in 3D Slicer 5.6.2. Feature extraction used Python 3.11.7, NumPy 1.26, scikit-image 0.22, and SimpleITK 2.3; texture definitions were compatible with PyRadiomics 3.0.1. Regions of interest were delineated separately on all views in which the lesion was visible. Spot-compression or magnification views were used only when standard craniocaudal and mediolateral oblique views did not permit reliable delineation.

The circularity-deficit irregularity score was I = 1 - 4piA/P^2, where A and P are ROI area and perimeter; values were truncated to 0-1. The study-defined contour serration score was S = 1 - P_hull/P, where P_hull is the convex-hull perimeter. This score was treated as a quantitative surrogate of serrated or spiculated boundary complexity and was not considered equivalent to the radiologist-assigned BI-RADS spiculated-margin descriptor.

Symmetric gray-level co-occurrence matrices used pixel distances of 1 and 2 and directions of 0 degrees, 45 degrees, 90 degrees, and 135 degrees, with averaging across distances and directions. GLCM texture entropy was -sum[p_ij log2(p_ij + epsilon)]. Normalized GLCM contrast was sum[(i-j)^2 p_ij]/(N_g-1)^2, with N_g = 64. Across views, irregularity and contour serration scores were summarized by the maximum, whereas entropy and normalized contrast were summarized by an ROI-area-weighted mean; a single visible view contributed its observed value.

### 2.5 Missing data handling

Missingness was assessed before modeling. In the full-data fit, numeric variables were imputed using cohort medians and unavailable categorical values were assigned to an Unknown category. During internal validation, imputation values and all subsequent preprocessing parameters were re-estimated within each bootstrap resample. Not applicable categories were retained as structured levels. The full-cohort imputation values and factor reference levels required to apply the final equation are reported in the **Supplementary Materials**.

### 2.6 Model development

Four models were evaluated. Model 1 used BI-RADS subcategory only. Model 2 combined the eight clinical candidate variables with BI-RADS subcategory. Model 3 used LASSO logistic regression with the full clinical and mammographic candidate set. Model 4 was a sensitivity LASSO model that used the same candidate set but excluded BI-RADS subcategory, thereby assessing information beyond the radiologist-assigned category. Ten-fold cross-validation selected the penalty parameter, and the one-standard-error penalty parameter (lambda.1se) was used to favor parsimony and reduce overfitting risk (20, 21).

### 2.7 Statistical analysis

Continuous variables were summarized as median and interquartile range, and categorical variables as frequency and percentage. Benign and malignant groups were compared using Wilcoxon rank-sum tests and chi-square or Fisher exact tests, as appropriate. Univariable logistic regression estimated odds ratios and 95% confidence intervals. For univariable presentation, age and lesion size were scaled per 5 units, and the quantitative contour and contrast scores per 0.1 increase; model development used their recorded continuous values.

Model performance was summarized by AUC, Brier score, calibration intercept, calibration slope, and a decile-based calibration plot with exact binomial 95% confidence intervals for observed malignancy rates (22). Internal validation used 1,000 paired patient-level bootstrap resamples. Within each resample, numeric imputation, categorical encoding, standardization, 10-fold cross-validation, penalty selection, LASSO feature selection, and coefficient estimation were repeated. Optimism-corrected estimates and bootstrap 95% confidence intervals were calculated for AUC, Brier score, calibration intercept, and calibration slope; model differences were estimated with the same paired resamples. Clinical utility was evaluated by decision curve analysis (23). The model-positive action was prioritization for expedited tissue diagnosis within the existing BI-RADS 4 biopsy pathway, not biopsy versus no biopsy. Net benefit was evaluated from 0.10 to 0.50; 0.10 and 0.50 corresponded to the BI-RADS 4A/4B and 4B/4C probability boundaries, whereas 0.20 and 0.30 were predefined intermediate scenarios. Within-category performance used bootstrap out-of-bag predictions for BI-RADS 4A, 4B, and 4C. A sensitivity analysis was restricted to histopathologically verified lesions. Prediction model reporting followed TRIPOD and TRIPOD+AI guidance (18, 19). Analyses were conducted in R version 4.5.3 (R Foundation for Statistical Computing, Vienna, Austria).

OpenAI ChatGPT (web version accessed July 2026; GPT-5.6 Thinking model; OpenAI, San Francisco, CA, United States) assisted with drafting and checking R code. All analyses were executed locally, and all code, outputs, and interpretations were reviewed and verified by the authors.

## 3 Results

### 3.1 Cohort selection

The verified screening cohort included 728 patients. After excluding 198 with BI-RADS category 3 or 5 findings, 18 with prior breast cancer treatment, 46 without an acceptable reference standard, and 25 with incomplete primary predictor data, 441 patients with one index BI-RADS 4 lesion each were included. All 441 unique patients contributed one analytic record; 165 lesions were malignant (**Figure 1**).

**Figure 1:**
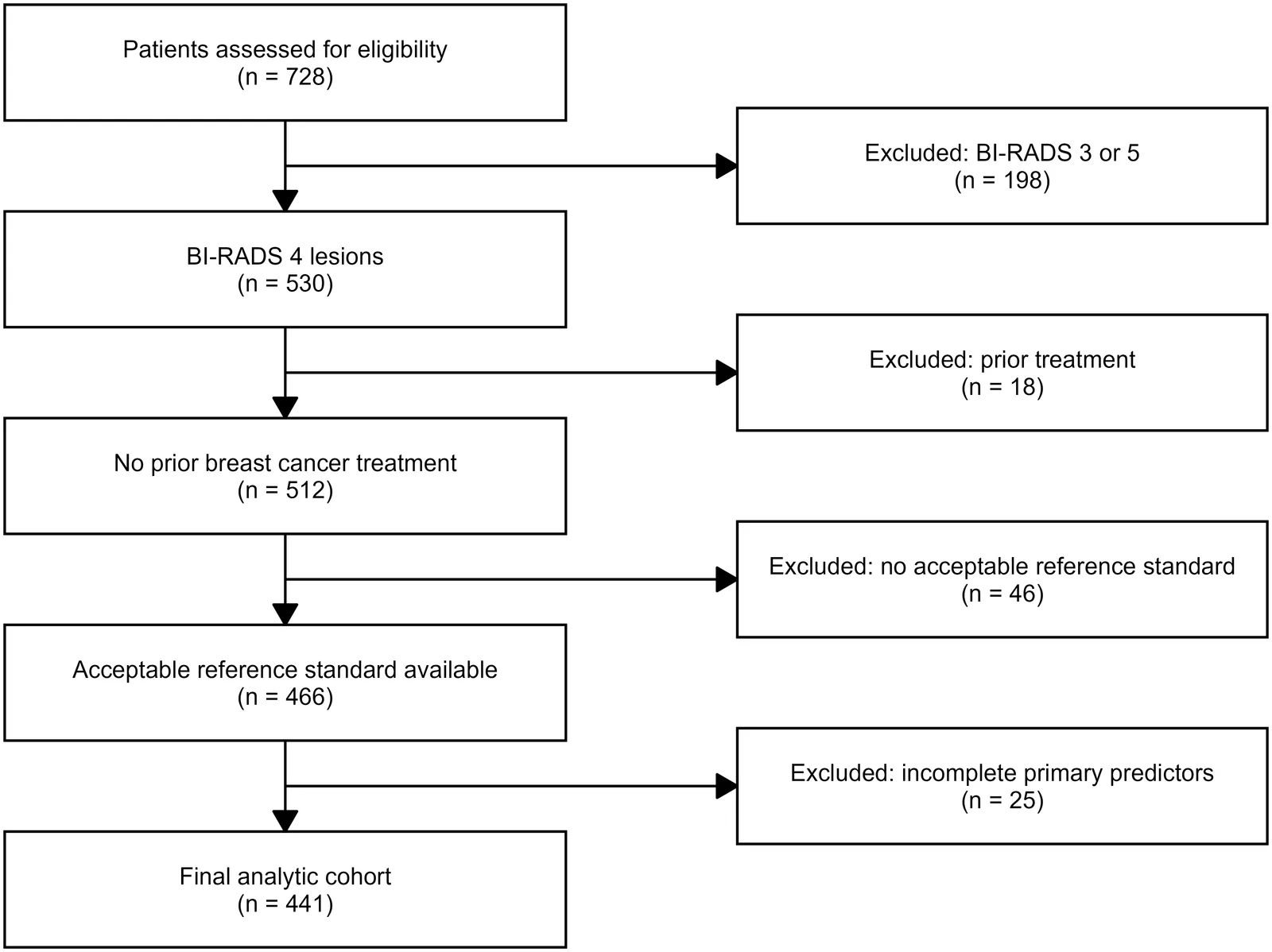
Study flow diagram. The verified screening cohort included 728 patients. After sequential exclusion of 198 patients with BI-RADS category 3 or 5 findings, 18 with prior breast cancer treatment, 46 without an acceptable reference standard, and 25 with incomplete primary predictor data, 441 patients with one index BI-RADS 4 lesion each were included.

### 3.2 Reference-standard composition

Among 276 benign lesions, 232 were histopathologically verified and 44 were classified as benign by longitudinal imaging stability. The latter included 34 BI-RADS 4A, nine BI-RADS 4B, and one BI-RADS 4C lesion and had a median follow-up of 34.5 months (interquartile range, 29.0-45.3). The analytic extract identified 110 malignant diagnoses verified by core-needle biopsy and 55 by surgery; the exact percutaneous or surgical procedure subtype for the 232 benign histopathologic diagnoses was not consistently retained.

### 3.3 Clinical and mammographic characteristics

Baseline clinical characteristics are summarized in **Table 1**. The median age was 49.0 years (interquartile range, 43.0-55.0 years). Age was slightly higher in the malignant group than in the benign group, but the difference did not reach statistical significance (50.0 vs. 48.0 years; P = 0.081). Postmenopausal status was more frequent in malignant lesions than in benign lesions (57.3% vs. 47.4%; P = 0.045). Palpable mass was also more common in the malignant group (41.0% vs. 29.4%; P = 0.014). Family history, body mass index, previous benign breast biopsy, diabetes, and hypertension were not significantly different between groups.

**Table 1:** Baseline clinical characteristics of BI-RADS 4 lesions by final malignancy outcome.

Characteristic	Overall (n=441)	Benign (n=276)	Malignant (n=165)	P value
Age, years	49.0 [43.0, 55.0]	48.0 [43.0, 55.0]	50.0 [44.0, 56.0]	0.081
Menopausal status				0.045
Premenopausal	214 (48.9%)	144 (52.6%)	70 (42.7%)	
Postmenopausal	224 (51.1%)	130 (47.4%)	94 (57.3%)	
First-degree family history of breast cancer				0.385
No	416 (95.2%)	258 (94.5%)	158 (96.3%)	
Yes	21 (4.8%)	15 (5.5%)	6 (3.7%)	
BMI, kg/m2	23.9 [22.0, 25.9]	23.8 [22.0, 25.8]	24.1 [21.9, 26.1]	0.617
Prior benign breast biopsy				0.246
No	374 (88.4%)	238 (89.8%)	136 (86.1%)	
Yes	49 (11.6%)	27 (10.2%)	22 (13.9%)	
Diabetes				0.225
No	416 (97.2%)	258 (96.3%)	158 (98.8%)	
Yes	12 (2.8%)	10 (3.7%)	2 (1.2%)	
Hypertension				0.955
No	388 (91.3%)	243 (91.4%)	145 (91.2%)	
Yes	37 (8.7%)	23 (8.6%)	14 (8.8%)	
Palpable mass				0.014
No	285 (66.3%)	190 (70.6%)	95 (59.0%)	
Yes	145 (33.7%)	79 (29.4%)	66 (41.0%)	

Values are median [interquartile range] for continuous variables and n (%) for categorical variables. Percentages were calculated among non-missing records. P values were calculated using Wilcoxon rank-sum tests for continuous variables and chi-square or Fisher exact tests for categorical variables as appropriate.

Mammographic characteristics are shown in **Table 2**. BI-RADS subcategory was strongly associated with malignancy (P < 0.001). Observed malignancy rates were 15.7% (34/216), 46.4% (70/151), and 82.4% (61/74) for BI-RADS 4A, 4B, and 4C, respectively (**Figure 2**). Malignant lesions were larger than benign lesions (median, 18.1 vs. 15.4 mm; P < 0.001). Irregular shape, suspicious margins, high relative density, calcification presence, larger calcification cluster extent, architectural distortion, and skin or nipple retraction were more frequent among malignant lesions. Circularity-deficit irregularity score, contour serration score, GLCM texture entropy, and normalized GLCM contrast were also higher in malignant lesions.

**Table 2:** Mammographic characteristics of BI-RADS 4 lesions by final malignancy outcome.

Characteristic	Overall (n=441)	Benign (n=276)	Malignant (n=165)	P value
BI-RADS subcategory				<0.001
BI-RADS 4A	216 (49.0%)	182 (65.9%)	34 (20.6%)	
BI-RADS 4B	151 (34.2%)	81 (29.3%)	70 (42.4%)	
BI-RADS 4C	74 (16.8%)	13 (4.7%)	61 (37.0%)	
Breast density				0.482
A	33 (7.5%)	20 (7.3%)	13 (7.9%)	
B	139 (31.7%)	94 (34.3%)	45 (27.3%)	
C	207 (47.2%)	123 (44.9%)	84 (50.9%)	
D	60 (13.7%)	37 (13.5%)	23 (13.9%)	
Dense breast				0.180
No	172 (39.2%)	114 (41.6%)	58 (35.2%)	
Yes	267 (60.8%)	160 (58.4%)	107 (64.8%)	
Lesion type				0.073
Architectural distortion	28 (6.3%)	13 (4.7%)	15 (9.1%)	
Asymmetry	39 (8.8%)	31 (11.2%)	8 (4.8%)	
Calcification	91 (20.6%)	58 (21.0%)	33 (20.0%)	
Mass	234 (53.1%)	146 (52.9%)	88 (53.3%)	
Mass with calcification	49 (11.1%)	28 (10.1%)	21 (12.7%)	
Lesion size, mm	16.3 [12.6, 21.2]	15.4 [12.1, 19.9]	18.1 [13.7, 23.3]	<0.001
Mass shape				0.002
Irregular	157 (36.0%)	82 (29.9%)	75 (46.3%)	
Not applicable	157 (36.0%)	101 (36.9%)	56 (34.6%)	
Oval	85 (19.5%)	64 (23.4%)	21 (13.0%)	
Round	37 (8.5%)	27 (9.9%)	10 (6.2%)	
Mass margin				<0.001
Circumscribed	93 (21.5%)	70 (26.1%)	23 (14.0%)	
Indistinct	45 (10.4%)	19 (7.1%)	26 (15.9%)	
Microlobulated	38 (8.8%)	20 (7.5%)	18 (11.0%)	
Not applicable	156 (36.1%)	100 (37.3%)	56 (34.1%)	
Obscured	67 (15.5%)	45 (16.8%)	22 (13.4%)	
Spiculated	33 (7.6%)	14 (5.2%)	19 (11.6%)	
Relative density				0.016
Equal	149 (34.6%)	91 (33.7%)	58 (36.0%)	
High	67 (15.5%)	33 (12.2%)	34 (21.1%)	
Low	59 (13.7%)	45 (16.7%)	14 (8.7%)	
Not applicable	156 (36.2%)	101 (37.4%)	55 (34.2%)	
Calcification present				0.025
No	252 (57.1%)	169 (61.2%)	83 (50.3%)	
Yes	189 (42.9%)	107 (38.8%)	82 (49.7%)	
Calcification cluster extent, mm	0.0 [0.0, 6.4]	0.0 [0.0, 4.0]	0.0 [0.0, 8.4]	0.003
Architectural distortion				0.001
No	382 (88.2%)	248 (92.2%)	134 (81.7%)	
Yes	51 (11.8%)	21 (7.8%)	30 (18.3%)	
Skin/nipple retraction				0.010
No	414 (95.4%)	263 (97.4%)	151 (92.1%)	
Yes	20 (4.6%)	7 (2.6%)	13 (7.9%)	
Suspicious axillary lymph nodes				0.116
No	406 (93.8%)	257 (95.2%)	149 (91.4%)	
Yes	27 (6.2%)	13 (4.8%)	14 (8.6%)	
Circularity-deficit irregularity score	0.41 [0.29, 0.52]	0.35 [0.25, 0.44]	0.51 [0.41, 0.60]	<0.001
Contour serration score	0.30 [0.20, 0.42]	0.24 [0.16, 0.36]	0.41 [0.30, 0.52]	<0.001
GLCM texture entropy	4.48 [4.15, 4.82]	4.38 [4.08, 4.81]	4.56 [4.26, 4.83]	0.003
Normalized GLCM contrast	0.48 [0.36, 0.60]	0.42 [0.32, 0.54]	0.56 [0.46, 0.69]	<0.001

Not applicable categories indicate descriptors that were not clinically applicable to the lesion type and were treated as structured categories rather than missing data.

**Figure 2:**
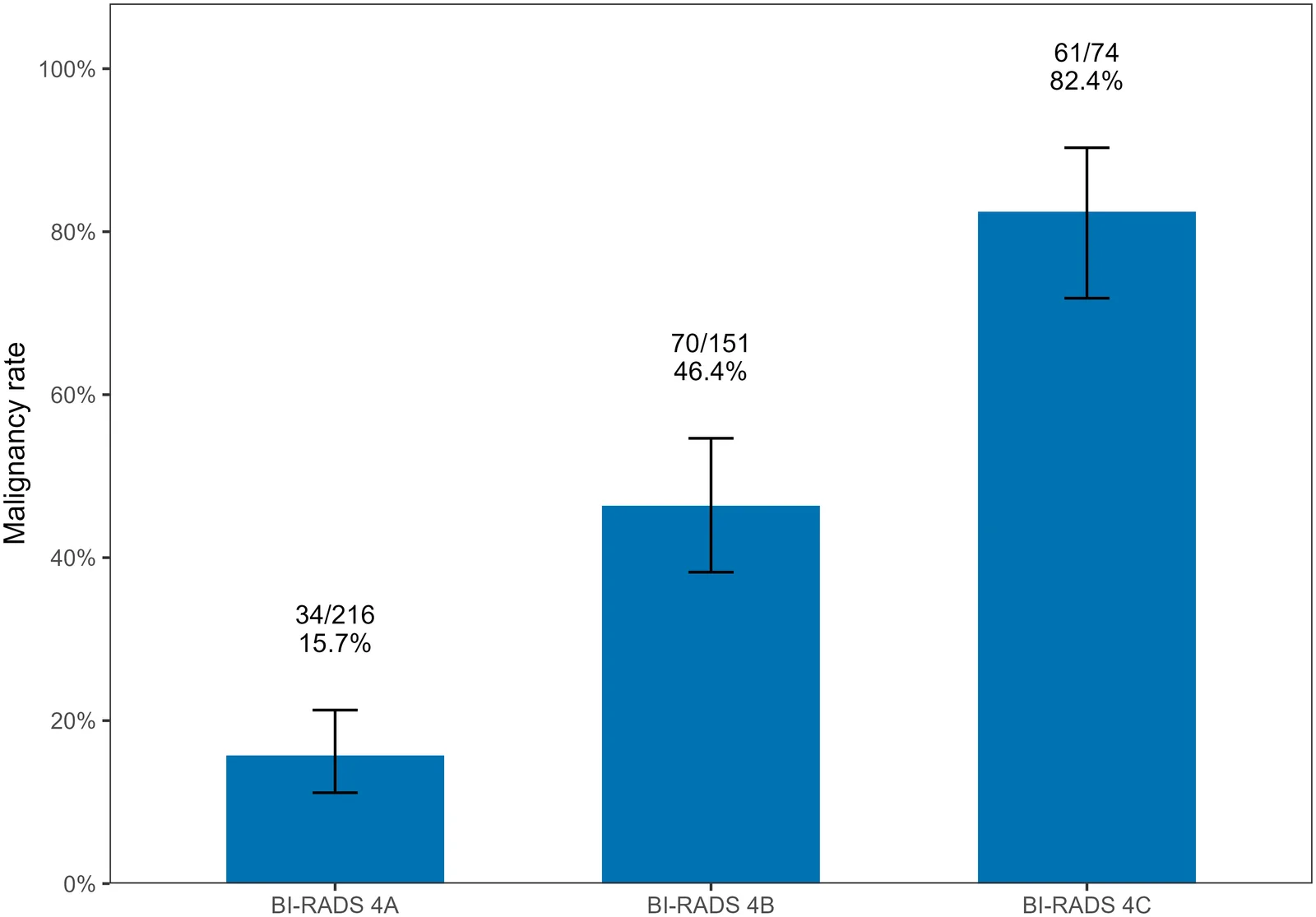
Malignancy rates across BI-RADS 4 subcategories. Bars show observed proportions and error bars show exact binomial 95% confidence intervals. Labels report malignant lesions/total lesions and percentages.

### 3.4 Univariable associations with malignancy

Univariable logistic regression results are presented in **Table 3**. Compared with BI-RADS 4A, BI-RADS 4B and 4C were associated with higher malignancy odds (OR, 4.63; 95% CI, 2.87-7.59; and OR, 25.1; 95% CI, 12.8-52.6; both P < 0.001). Lesion size was associated with malignancy per 5-mm increase (OR, 1.28; 95% CI, 1.12-1.46; P < 0.001). Suspicious mass margins, calcification presence and extent, architectural distortion, skin or nipple retraction, circularity-deficit irregularity score, contour serration score, GLCM texture entropy, and normalized GLCM contrast were also associated with malignancy.

**Table 3:** Univariable associations with malignancy in BI-RADS 4 lesions.

Characteristic	N	OR	95% CI	P value
Age, per 5 years	441	1.09	0.98, 1.22	0.115
Menopausal status	441			
Premenopausal		–	–	
Postmenopausal		1.49	1.01, 2.20	0.046
Unknown		1.03	0.05, 10.9	0.982
Palpable mass	441			
No		–	–	
Yes		1.67	1.11, 2.52	0.014
Unknown		1.14	0.29, 3.88	0.835
BI-RADS subcategory	441			
BI-RADS 4A		–	–	
BI-RADS 4B		4.63	2.87, 7.59	<0.001
BI-RADS 4C		25.1	12.8, 52.6	<0.001
Lesion size, per 5 mm	441	1.28	1.12, 1.46	<0.001
Mass shape	441			
Irregular		–	–	
Not applicable		0.61	0.38, 0.95	0.030
Oval		0.36	0.20, 0.64	<0.001
Round		0.40	0.18, 0.87	0.025
Mass margin	441			
Circumscribed		–	–	
Indistinct		4.16	1.97, 9.01	<0.001
Microlobulated		2.74	1.24, 6.10	0.013
Not applicable		1.70	0.97, 3.06	0.068
Obscured		1.49	0.74, 2.99	0.262
Spiculated		4.13	1.81, 9.71	<0.001
Relative density	441			
Equal		–	–	
High		1.62	0.90, 2.90	0.105
Low		0.49	0.24, 0.95	0.040
Not applicable		0.85	0.54, 1.36	0.507
Calcification present	441			
No		–	–	
Yes		1.56	1.06, 2.31	0.025
Calcification cluster extent, per 5 mm	441	1.34	1.12, 1.62	0.002
Architectural distortion	441			
No		–	–	
Yes		2.64	1.46, 4.85	0.001
Skin/nipple retraction	441			
No		–	–	
Yes		3.23	1.30, 8.77	0.014
Suspicious axillary lymph nodes	441			
No		–	–	
Yes		1.86	0.85, 4.11	0.120
Circularity-deficit irregularity score, per 0.1 increase	441	2.05	1.74, 2.46	<0.001
Contour serration score, per 0.1 increase	441	2.00	1.70, 2.36	<0.001
GLCM texture entropy, per 0.5 increase	441	1.39	1.13, 1.71	0.002
Normalized GLCM contrast, per 0.1 increase	441	1.80	1.56, 2.10	<0.001

Odds ratios were estimated using univariable logistic regression. Continuous variables were rescaled to clinically interpretable units before modeling. CI, confidence interval; OR, odds ratio.

### 3.5 Final LASSO model

At lambda.1se, the final full mammography-based LASSO model retained BI-RADS 4B, BI-RADS 4C, architectural distortion, circularity-deficit irregularity score, contour serration score, and normalized GLCM contrast (**Table 4**). Bootstrap selection frequencies were 98.3%, 100.0%, 69.9%, 99.3%, 99.9%, and 89.6%, respectively. No clinical variable was retained. The complete equation, factor reference levels, imputation values, and selection-stability results are provided in **Table 4** and the **Supplementary Materials**.

**Table 4:** Final full mammography-based LASSO model equation and coefficient stability.

Predictor	Coding/reference	Full-data coefficient	Bootstrap coefficient interval	Selection frequency
Intercept	Not applicable	-3.443663	-6.102 to -2.929	100.0%
BI-RADS 4B	Reference: BI-RADS 4A	0.505122	0.069 to 1.259	98.3%
BI-RADS 4C	Reference: BI-RADS 4A	1.776109	1.296 to 2.723	100.0%
Architectural distortion	Yes vs. No	0.075486	0.000 to 1.011	69.9%
Circularity-deficit irregularity score	Per 1.0 recorded-unit increase	2.345170	0.494 to 4.733	99.3%
Contour serration score	Per 1.0 recorded-unit increase	2.847925	1.282 to 4.783	99.9%
Normalized GLCM contrast	Per 1.0 recorded-unit increase	0.995985	0.000 to 2.768	89.6%

The final lambda.1se model was fitted with alpha = 1. Coefficients are on the original predictor scale; categorical terms use indicator coding. Bootstrap intervals are the 2.5th and 97.5th percentiles across 1,000 complete-pipeline refits and include zero when a term was not selected. LP = -3.443663 + 0.505122(BI-RADS 4B) + 1.776109(BI-RADS 4C) + 0.075486(architectural distortion) + 2.345170(irregularity score) + 2.847925(contour serration score) + 0.995985(normalized GLCM contrast); probability = exp(LP)/[1 + exp(LP)].

### 3.6 Model performance and internal validation

Model performance is summarized in **Table 5** and **Figure 3**. Optimism-corrected AUCs were 0.770 (95% CI, 0.729-0.815) for Model 1, 0.774 (0.733-0.814) for Model 2, 0.841 (0.809-0.877) for Model 3, and 0.766 (0.726-0.809) for Model 4. Corrected Brier scores were 0.177 (0.159-0.198), 0.183 (0.164-0.205), 0.160 (0.142-0.177), and 0.194 (0.177-0.214), respectively. Clinical variables did not improve Model 2 over BI-RADS alone: the corrected AUC difference was 0.005 (95% CI, -0.028 to 0.028), and the corrected Brier difference was 0.006 (-0.003 to 0.018).

**Table 5:** Apparent and optimism-corrected performance of the candidate models.

Model	Apparent AUC	Corrected AUC (95% CI)	Apparent Brier	Corrected Brier (95% CI)	Corrected intercept (95% CI)	Corrected slope (95% CI)	Bootstrap n
Model 1: BI-RADS-only	0.771	0.770 (0.729-0.815)	0.174	0.177 (0.159-0.198)	0.003 (-0.224 to 0.218)	0.986 (0.791-1.204)	1000
Model 2: Clinical + BI-RADS	0.805	0.774 (0.733-0.814)	0.168	0.183 (0.164-0.205)	0.008 (-0.231 to 0.233)	0.737 (0.387-1.003)	1000
Model 3: Full mammography-based LASSO	0.863	0.841 (0.809-0.877)	0.148	0.160 (0.142-0.177)	0.002 (-0.209 to 0.208)	1.445 (1.140-1.753)	1000
Model 4: LASSO without BI-RADS	0.796	0.766 (0.726-0.809)	0.182	0.194 (0.177-0.214)	0.001 (-0.205 to 0.206)	1.548 (1.268-1.876)	1000

Corrected estimates and 95% confidence intervals were obtained from 1,000 paired patient-level bootstrap resamples in which the complete preprocessing, tuning, selection, and estimation pipeline was repeated. Model 4 excluded BI-RADS subcategory.

**Figure 3:**
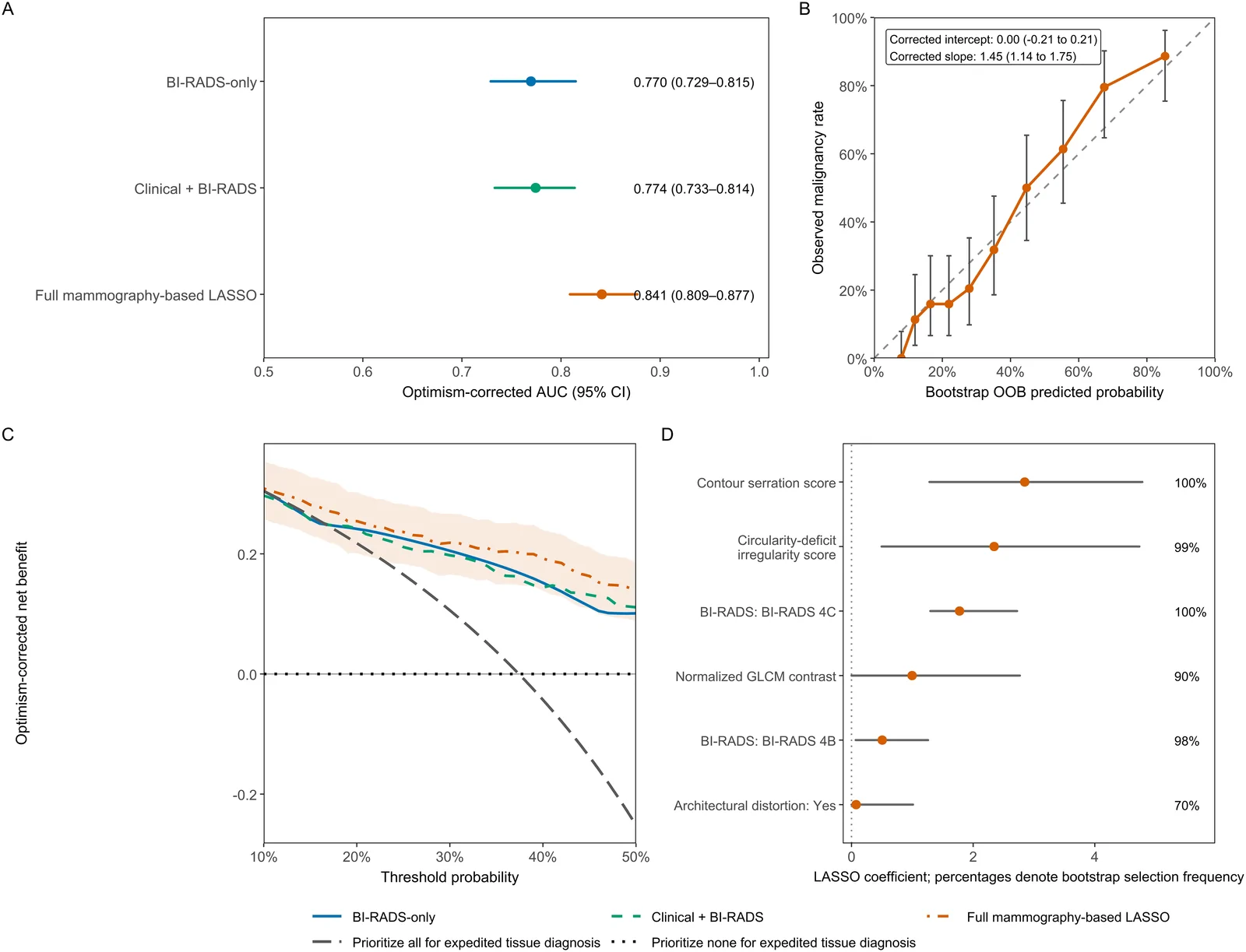
Internal validation, calibration, clinical utility, and model stability. **(A)** Optimism-corrected AUCs and 95% confidence intervals from 1,000 paired patient-level bootstrap resamples. **(B)** Calibration of the full mammography-based LASSO model using bootstrap out-of-bag predictions grouped into deciles; points show observed malignancy rates, error bars show exact binomial 95% confidence intervals, and the diagonal indicates perfect calibration. **(C)** Optimism-corrected decision curves for prioritization of expedited tissue diagnosis across thresholds of 0.10-0.50; the shaded band is the 95% bootstrap interval for the full model. Model positivity does not indicate biopsy versus no biopsy, and all BI-RADS 4 lesions remained candidates for tissue diagnosis. **(D)** Coefficients from the final lambda.1se model; horizontal bars show bootstrap 2.5th-97.5th percentile intervals, and percentages show the frequency of a nonzero coefficient across 1,000 bootstrap resamples. Continuous predictors are shown on the original scale used in the prediction equation. BI-RADS, Breast Imaging Reporting and Data System; GLCM, gray-level co-occurrence matrix; OOB, out-of-bag.

Model 3 improved corrected AUC versus Model 1 by 0.072 (95% CI, 0.028-0.108) and versus Model 2 by 0.067 (0.029-0.103). The corrected Brier difference was -0.018 (-0.033 to 0.002) versus Model 1 and -0.023 (-0.040 to -0.004) versus Model 2. Model 3 also outperformed the model excluding BI-RADS, with a corrected AUC difference of 0.075 (0.047-0.097) and Brier difference of -0.035 (-0.045 to -0.022). The corrected calibration intercept for Model 3 was 0.002 (95% CI, -0.209 to 0.208), whereas the slope was 1.445 (1.140-1.753), indicating under-dispersed predicted probabilities.

Using bootstrap out-of-bag predictions, Model 3 retained within-category discrimination: AUCs were 0.785 in BI-RADS 4A, 0.721 in BI-RADS 4B, and 0.758 in BI-RADS 4C. In the histopathology-only cohort of 397 patients, the corrected AUC for Model 3 was 0.835 (95% CI, 0.799-0.871); corrected AUC differences versus Models 1 and 2 were 0.073 (0.024-0.113) and 0.067 (0.018-0.109), respectively.

Decision curve analysis showed the highest corrected net benefit for Model 3 across the evaluated 0.10-0.50 threshold range (**Figure 3**). At thresholds of 0.10, 0.20, 0.30, and 0.50, the model prioritized 401, 301, 227, and 131 lesions for expedited tissue diagnosis and did not prioritize 0, 14, 26, and 64 malignant lesions, respectively. These thresholds were decision scenarios within the existing biopsy pathway and were not interpreted as rules for withholding tissue diagnosis.

## 4 Discussion

In this single-center retrospective diagnostic prediction study, a full mammography-based LASSO model improved internally validated discrimination beyond BI-RADS subcategory alone and beyond clinical variables plus BI-RADS. The corrected AUC was 0.841, and the improvement over the BI-RADS-only model was 0.072. The pathology-only analysis and within-subcategory analyses supported the robustness and incremental ranking value of the mammographic features.

The observed risk gradient across BI-RADS 4A, 4B, and 4C was clinically plausible. Malignancy rates increased from 15.7% in BI-RADS 4A to 46.4% in BI-RADS 4B and 82.4% in BI-RADS 4C. This pattern is consistent with the BI-RADS concept that category 4 covers a broad risk interval and requires subcategorization to communicate the degree of suspicion (5). The 4A malignancy rate in this cohort was higher than the lower-risk range commonly expected for 4A lesions, probably reflecting the diagnostic and referral-enriched nature of a tertiary hospital cohort rather than a population screening setting (11, 12). Accordingly, the model should be interpreted as a tool for a diagnostic BI-RADS 4 population, not as a screening-population risk calculator.

The retained predictors were consistent with established radiological interpretation. BI-RADS 4C and 4B remained central to risk estimation. The model additionally retained the study-defined contour serration score, circularity-deficit irregularity score, and normalized GLCM contrast, consistent with evidence linking irregular morphology and suspicious margins to malignancy on mammography (7–9). Architectural distortion had a small full-data coefficient and a 69.9% bootstrap selection frequency; its contribution should therefore be interpreted cautiously, although its direction was compatible with the clinical suspicion associated with this descriptor (6).

A practical feature of this study is the use of structured mammographic information rather than a purely black-box image model. Large mammography studies have shown that artificial intelligence can support cancer detection in selected settings (24, 25). More recently, a mammography-based deep learning model focusing on BI-RADS 4 lesions reported potential for reducing unnecessary biopsies (17). However, deployment of deep learning models can be limited by access to raw images, vendor-specific preprocessing, data governance, and external generalizability. A model based on structured report variables and transparent quantitative descriptors is easier to audit and explain during clinical review.

Our findings are also consistent with recent BI-RADS 4 prediction-model work using ultrasound, elastography, or automated breast ultrasound. Yang and colleagues developed a nomogram for BI-RADS 4A lesions in dense breasts, and Ren and colleagues reported a CEUS/SWE-based nomogram with good discrimination for BI-RADS 4 lesions (14, 15). Jiang and colleagues showed that a nomogram incorporating automated breast ultrasound features could improve diagnosis of BI-RADS 4 breast nodules (16). Compared with these studies, our model focuses on mammography-derived structured descriptors, which may be useful in institutions where mammography is the first-line diagnostic record and raw-image model deployment is not routine.

The clinical interpretation should remain conservative. BI-RADS 4 lesions generally warrant tissue diagnosis, and the model should not be used to replace or cancel biopsy (5). The intended role is risk communication and prioritization for expedited tissue diagnosis within the existing biopsy pathway; lesions below a threshold remain candidates for standard tissue sampling. Decision curve analysis evaluates net benefit across threshold probabilities rather than discrimination alone (23).

Calibration also limited probability-level interpretation. Model 3 had an optimism-corrected calibration intercept of 0.002, but its slope was 1.445, indicating under-dispersion: low predictions tended to be too high and high predictions too low. Calibration is essential because clinical actions depend on absolute probabilities rather than rank ordering alone (26). Accordingly, the model should be interpreted primarily as an internally validated risk-stratification tool; individual probabilities and decision thresholds require external validation and, if necessary, recalibration.

The validated results did not support incremental value from the clinical variables in Model 2: its corrected AUC was essentially unchanged from BI-RADS alone, its corrected Brier score was numerically worse, and its calibration slope was lower. Conversely, excluding BI-RADS from the LASSO model reduced performance. Together, these findings indicate that BI-RADS remains indispensable, while the principal incremental information came from lesion-level quantitative and structured mammographic features.

From a methodological perspective, LASSO logistic regression was suitable for a moderate-sized candidate predictor set, but it does not eliminate overfitting risk. Penalized regression can shrink coefficients and select a parsimonious predictor set, yet model stability still depends on sample size, event count, predictor correlation, and case mix (20, 27). The complete-pipeline paired bootstrap repeated preprocessing, tuning, selection, and estimation within each resample, reducing information leakage and providing selection-frequency estimates. Internal validation nevertheless does not replace temporal or external validation (28). Future work should test the model in independent hospitals and in cohorts with different biopsy thresholds, breast density distributions, and reporting practices.

This study has several strengths. First, it addresses the clinically heterogeneous BI-RADS 4 category. Second, the model uses transparent, auditable mammographic descriptors. Third, evaluation included complete-pipeline paired bootstrap correction, paired performance differences, calibration with uncertainty, within-BI-RADS analyses, pathology-only sensitivity analysis, and decision curve analysis (18, 29). Fourth, the complete equation, reference levels, imputation rules, and bootstrap selection frequencies improve reproducibility.

The study also has limitations. It was retrospective and conducted at a single tertiary hospital, so selection and case-mix effects are likely. Forty-four benign lesions were classified by longitudinal imaging stability rather than histopathology, creating potential differential verification despite consistent pathology-only results. The study did not include external validation, which remains the principal barrier to clinical implementation and an important prediction-model risk-of-bias consideration (30). The extracted database did not consistently retain the exact core-needle biopsy, vacuum-assisted biopsy, or surgical procedure subtype for the 232 benign histopathologic diagnoses. Formal inter-reader and intra-reader reproducibility of manual segmentation and quantitative feature extraction was not evaluated; observer variability is recognized in BI-RADS reporting (9). The quantitative features are institution-specific and require cross-reader, cross-software, and cross-system validation. Finally, the calibration slope indicated under-dispersed probabilities, and the evaluated decision thresholds were exploratory rather than validated clinical cutoffs.

In summary, the model improved risk ranking within BI-RADS 4 lesions but did not establish a safe biopsy-withholding strategy. Its most defensible role is research-stage risk communication and prioritization for expedited tissue diagnosis, pending independent validation and calibration assessment.

## 5 Conclusion

The full mammography-based LASSO model improved internally validated discrimination and within-subcategory risk stratification among BI-RADS 4 lesions. Clinical variables did not add validated predictive value, and probability calibration remained under-dispersed. The model should not replace tissue diagnosis and requires external validation and potential recalibration before clinical use.

## Data Availability

The original contributions presented in the study are included in the article/**Supplementary Material**. Further inquiries can be directed to the corresponding author.
