# Supplementary material for: Development and internal validation of an interpretable mammography-based model for malignancy risk stratification in BI-RADS 4 breast lesions
Source: Front Oncol. 2026 Oct 26;16:1900262. doi: 10.3389/fonc.2026.1900262 (PMC13745337; doi:10.3389/fonc.2026.1900262)
Supplement: Supplementary file 1 [file DataSheet1.docx]

**Supplementary Materials**

**1. Supplementary Tables**

**Supplementary Table S1. Missingness of candidate predictors.**

Percentages are based on the 441-patient primary cohort. Numeric imputation and categorical missing-level encoding were repeated within every bootstrap resample.

| **Variable** | **Missing, n** | **Total, n** | **Missing, %** |
| --- | --- | --- | --- |
| GLCM texture entropy | 46 | 441 | 10.4 |
| Contour serration score | 42 | 441 | 9.5 |
| Normalized GLCM contrast | 38 | 441 | 8.6 |
| Circularity-deficit irregularity score | 36 | 441 | 8.2 |
| Body mass index, kg/m² | 21 | 441 | 4.8 |
| Calcification cluster extent, mm | 20 | 441 | 4.5 |
| Prior benign breast biopsy | 18 | 441 | 4.1 |
| Hypertension | 16 | 441 | 3.6 |
| Diabetes | 13 | 441 | 2.9 |
| Palpable mass | 11 | 441 | 2.5 |
| Relative density | 10 | 441 | 2.3 |
| Lesion size, mm | 9 | 441 | 2.0 |
| Mass margin | 9 | 441 | 2.0 |
| Architectural distortion | 8 | 441 | 1.8 |
| Suspicious axillary lymph nodes | 8 | 441 | 1.8 |
| Skin or nipple retraction | 7 | 441 | 1.6 |
| Mass shape | 5 | 441 | 1.1 |
| First-degree family history of breast cancer | 4 | 441 | 0.9 |
| Asymmetry | 3 | 441 | 0.7 |
| Menopausal status | 3 | 441 | 0.7 |
| Breast density | 2 | 441 | 0.5 |
| Age, years | 0 | 441 | 0.0 |
| BI-RADS 4 subcategory | 0 | 441 | 0.0 |
| Calcification number category | 0 | 441 | 0.0 |
| Calcification present | 0 | 441 | 0.0 |
| Lesion type | 0 | 441 | 0.0 |

**Supplementary Table S2. Candidate predictor dictionary and excluded variables.**

| **Domain** | **Variable** | **Eligible model(s)** | **Rationale/handling** |
| --- | --- | --- | --- |
| Clinical | Age | Model 2 and Model 3 | Available at index mammography |
| Clinical | Menopausal status | Model 2 and Model 3 | Available at index mammography |
| Clinical | First-degree family history of breast cancer | Model 2 and Model 3 | Available at index mammography |
| Clinical | Body mass index | Model 2 and Model 3 | Available at index mammography |
| Clinical | Prior benign breast biopsy | Model 2 and Model 3 | Available at index mammography |
| Clinical | Diabetes | Model 2 and Model 3 | Available at index mammography |
| Clinical | Hypertension | Model 2 and Model 3 | Available at index mammography |
| Clinical | Palpable mass | Model 2 and Model 3 | Available at index mammography |
| Mammographic | BI-RADS 4 subcategory | Model 3 | Available at index mammography |
| Mammographic | Breast density | Model 3 | Available at index mammography |
| Mammographic | Lesion type | Model 3 | Available at index mammography |
| Mammographic | Lesion size | Model 3 | Available at index mammography |
| Mammographic | Mass shape | Model 3 | Available at index mammography |
| Mammographic | Mass margin | Model 3 | Available at index mammography |
| Mammographic | Relative density | Model 3 | Available at index mammography |
| Mammographic | Calcification presence | Model 3 | Available at index mammography |
| Mammographic | Calcification number category | Model 3 | Available at index mammography |
| Mammographic | Calcification cluster extent | Model 3 | Available at index mammography |
| Mammographic | Architectural distortion | Model 3 | Available at index mammography |
| Mammographic | Asymmetry | Model 3 | Available at index mammography |
| Mammographic | Skin or nipple retraction | Model 3 | Available at index mammography |
| Mammographic | Suspicious axillary lymph nodes | Model 3 | Available at index mammography |
| Mammographic | Circularity-deficit irregularity score | Model 3 | Available at index mammography |
| Mammographic | Contour serration score | Model 3 | Available at index mammography |
| Mammographic | GLCM texture entropy | Model 3 | Available at index mammography |
| Mammographic | Normalized GLCM contrast | Model 3 | Available at index mammography |
| Excluded/descriptive | birads_score | No | Duplicate numerical encoding of BI-RADS subcategory |
| Excluded/descriptive | dense_breast | No | Binary derivative of four-level breast density |
| Excluded/descriptive | lesion_side | No | Anatomical localization variable rather than a stable lesion-level diagnostic predictor |
| Excluded/descriptive | lesion_quadrant | No | Anatomical localization variable with limited transportability |
| Excluded/descriptive | mammography_views | No | Workflow variable that may encode diagnostic work-up intensity |
| Excluded/descriptive | calcification_morphology | No | Conditionally applicable descriptor with greater unavailable information |
| Excluded/descriptive | calcification_distribution | No | Conditionally applicable descriptor with greater unavailable information |

**Supplementary Table S3. Definitions and aggregation of quantitative mammographic features.**

| **Variable** | **Publication label** | **Definition** | **Range or setting** | **Multiview aggregation** |
| --- | --- | --- | --- | --- |
| lesion_irregularity_score | Circularity-deficit irregularity score | 1 - 4*pi*A/P^2, where A is ROI area and P is ROI perimeter | Truncated to 0-1 | Maximum across visible views |
| spiculation_score | Contour serration score | 1 - P_hull/P, where P_hull is convex-hull perimeter and P is ROI perimeter | Study-defined quantitative contour measure; not equivalent to the BI-RADS spiculated-margin descriptor | Maximum across visible views |
| texture_entropy | GLCM texture entropy | -sum[p_ij*log2(p_ij + epsilon)] | 64 gray levels; distances 1 and 2 pixels; directions 0, 45, 90, and 135 degrees; symmetric matrices; averaged across distances and directions | ROI-area-weighted mean across visible views |
| lesion_contrast_score | Normalized GLCM contrast | sum[(i-j)^2*p_ij]/(N_g-1)^2, with N_g = 64 | Theoretical normalized range 0-1 under the stated discretization | ROI-area-weighted mean across visible views |

**Supplementary Table S4. Reference-standard composition.**

Surgical pathology superseded earlier percutaneous biopsy when available. Core-needle biopsy used a 14-gauge needle with four to six cores; vacuum-assisted biopsy used a 9-gauge or 11-gauge device.

| **Reference-standard category** | **N** | **Details** |
| --- | --- | --- |
| Histopathology | 397 | 110 malignant core-needle biopsy; 55 malignant surgical pathology; 232 benign histopathologic diagnoses. Exact benign procedure subtype was not consistently retained. |
| Longitudinal imaging stability | 44 | 34 BI-RADS 4A, nine 4B, one 4C; median 34.5 months (IQR 29.0-45.3; range 24.0-60.0). |

**Supplementary Table S5. Factor reference levels used in the prediction equation.**

| **Variable** | **Reference level** | **Unavailable-information level** |
| --- | --- | --- |
| Menopausal status | Premenopausal | Unknown |
| First-degree family history of breast cancer | No | Unknown |
| Prior benign breast biopsy | No | Unknown |
| Diabetes | No | Unknown |
| Hypertension | No | Unknown |
| Palpable mass | No | Unknown |
| BI-RADS 4 subcategory | BI-RADS 4A | Unknown |
| Breast density | A | Unknown |
| Lesion type | Mass | Unknown |
| Mass shape | Not_applicable | Unknown |
| Mass margin | Not_applicable | Unknown |
| Relative density | Not_applicable | Unknown |
| Calcification present | No | Unknown |
| Calcification number category | 0 | Unknown |
| Architectural distortion | No | Unknown |
| Asymmetry | No | Unknown |
| Skin or nipple retraction | No | Unknown |
| Suspicious axillary lymph nodes | No | Unknown |

**Supplementary Table S6. Numeric imputation values for application of the full-data equation.**

During bootstrap validation, medians were re-estimated separately within each resample.

| **Variable** | **Full-cohort median imputation value** |
| --- | --- |
| Age, years | 49.0 |
| Body mass index, kg/m² | 23.9 |
| Lesion size, mm | 16.3 |
| Calcification cluster extent, mm | 0.0 |
| Circularity-deficit irregularity score | 0.412 |
| Contour serration score | 0.299 |
| GLCM texture entropy | 4.48 |
| Normalized GLCM contrast | 0.484 |

**Supplementary Table S7. Paired optimism-corrected differences between models in the primary cohort.**

| **Metric** | **Comparison** | **Corrected difference** | **95% CI lower** | **95% CI upper** | **Successful bootstrap replicates** |
| --- | --- | --- | --- | --- | --- |
| AUC | M2 minus M1 | 0.005 | -0.028 | 0.028 | 1000 |
| Brier | M2 minus M1 | 0.006 | -0.003 | 0.018 | 1000 |
| Calibration_intercept | M2 minus M1 | 0.005 | -0.060 | 0.074 | 1000 |
| Calibration_slope | M2 minus M1 | -0.249 | -0.644 | -0.031 | 1000 |
| AUC | M3 minus M1 | 0.072 | 0.028 | 0.108 | 1000 |
| Brier | M3 minus M1 | -0.018 | -0.033 | 0.002 | 1000 |
| Calibration_intercept | M3 minus M1 | -0.001 | -0.095 | 0.094 | 1000 |
| Calibration_slope | M3 minus M1 | 0.459 | 0.159 | 0.742 | 1000 |
| AUC | M3 minus M2 | 0.067 | 0.029 | 0.103 | 1000 |
| Brier | M3 minus M2 | -0.023 | -0.040 | -0.004 | 1000 |
| Calibration_intercept | M3 minus M2 | -0.006 | -0.109 | 0.104 | 1000 |
| Calibration_slope | M3 minus M2 | 0.708 | 0.352 | 1.108 | 1000 |
| AUC | M4 minus M1 | -0.004 | -0.057 | 0.051 | 1000 |
| Brier | M4 minus M1 | 0.017 | -0.007 | 0.041 | 1000 |
| Calibration_intercept | M4 minus M1 | -0.002 | -0.144 | 0.128 | 1000 |
| Calibration_slope | M4 minus M1 | 0.562 | 0.230 | 0.918 | 1000 |
| AUC | M3 minus M4 | 0.075 | 0.047 | 0.097 | 1000 |
| Brier | M3 minus M4 | -0.035 | -0.045 | -0.022 | 1000 |
| Calibration_intercept | M3 minus M4 | 0.001 | -0.067 | 0.074 | 1000 |
| Calibration_slope | M3 minus M4 | -0.103 | -0.378 | 0.104 | 1000 |

**Supplementary Table S8. Model performance within BI-RADS 4 subcategories using bootstrap out-of-bag predictions.**

| **birads clean** | **Model** | **N** | **Malignant n** | **Malignancy rate** | **AUC** | **Brier** | **Calibration intercept** | **Calibration slope** |
| --- | --- | --- | --- | --- | --- | --- | --- | --- |
| BI-RADS 4A | M2 | 216 | 34 | 0.157 | 0.506 | 0.137 | -0.042 | -0.396 |
| BI-RADS 4A | M3 | 216 | 34 | 0.157 | 0.785 | 0.116 | -0.444 | 1.344 |
| BI-RADS 4A | M4 | 216 | 34 | 0.157 | 0.797 | 0.134 | -1.014 | 1.321 |
| BI-RADS 4B | M2 | 151 | 70 | 0.464 | 0.513 | 0.259 | 0.021 | -0.081 |
| BI-RADS 4B | M3 | 151 | 70 | 0.464 | 0.721 | 0.216 | 0.229 | 1.036 |
| BI-RADS 4B | M4 | 151 | 70 | 0.464 | 0.715 | 0.218 | 0.272 | 0.937 |
| BI-RADS 4C | M2 | 74 | 61 | 0.824 | 0.404 | 0.174 | 0.136 | -0.906 |
| BI-RADS 4C | M3 | 74 | 61 | 0.824 | 0.758 | 0.129 | 0.520 | 1.341 |
| BI-RADS 4C | M4 | 74 | 61 | 0.824 | 0.787 | 0.237 | 1.828 | 1.363 |

**Supplementary Table S9. Performance in the histopathology-only sensitivity cohort (n = 397; 165 malignant).**

| **Model** | **Metric** | **Apparent** | **Corrected estimate** | **95% CI lower** | **95% CI upper** | **Bootstrap n** |
| --- | --- | --- | --- | --- | --- | --- |
| Model 1: BI-RADS-only | AUC | 0.762 | 0.762 | 0.718 | 0.806 | 1000 |
| Model 1: BI-RADS-only | Brier | 0.184 | 0.187 | 0.170 | 0.208 | 1000 |
| Model 1: BI-RADS-only | Calibration_intercept | 0.000 | 0.003 | -0.226 | 0.239 | 1000 |
| Model 1: BI-RADS-only | Calibration_slope | 1.000 | 0.989 | 0.776 | 1.231 | 1000 |
| Model 2: Clinical + BI-RADS | AUC | 0.801 | 0.768 | 0.727 | 0.811 | 1000 |
| Model 2: Clinical + BI-RADS | Brier | 0.176 | 0.193 | 0.172 | 0.215 | 1000 |
| Model 2: Clinical + BI-RADS | Calibration_intercept | -0.000 | 0.007 | -0.257 | 0.256 | 1000 |
| Model 2: Clinical + BI-RADS | Calibration_slope | 1.000 | 0.704 | 0.330 | 0.993 | 1000 |
| Model 3: Full mammography-based LASSO | AUC | 0.861 | 0.835 | 0.799 | 0.871 | 1000 |
| Model 3: Full mammography-based LASSO | Brier | 0.155 | 0.169 | 0.152 | 0.190 | 1000 |
| Model 3: Full mammography-based LASSO | Calibration_intercept | -0.000 | 0.004 | -0.201 | 0.229 | 1000 |
| Model 3: Full mammography-based LASSO | Calibration_slope | 1.745 | 1.503 | 1.161 | 1.844 | 1000 |
| Model 4: LASSO without BI-RADS | AUC | 0.796 | 0.764 | 0.721 | 0.806 | 1000 |
| Model 4: LASSO without BI-RADS | Brier | 0.188 | 0.202 | 0.186 | 0.224 | 1000 |
| Model 4: LASSO without BI-RADS | Calibration_intercept | -0.000 | 0.006 | -0.195 | 0.225 | 1000 |
| Model 4: LASSO without BI-RADS | Calibration_slope | 1.893 | 1.644 | 1.335 | 1.971 | 1000 |

**Supplementary Table S10. Paired model differences in the histopathology-only cohort.**

| **Metric** | **Comparison** | **Corrected difference** | **95% CI lower** | **95% CI upper** | **Bootstrap n** |
| --- | --- | --- | --- | --- | --- |
| AUC | M2 minus M1 | 0.006 | -0.031 | 0.035 | 1000 |
| Brier | M2 minus M1 | 0.006 | -0.006 | 0.021 | 1000 |
| Calibration_intercept | M2 minus M1 | 0.004 | -0.070 | 0.088 | 1000 |
| Calibration_slope | M2 minus M1 | -0.285 | -0.696 | -0.031 | 1000 |
| AUC | M3 minus M1 | 0.073 | 0.024 | 0.113 | 1000 |
| Brier | M3 minus M1 | -0.019 | -0.037 | 0.004 | 1000 |
| Calibration_intercept | M3 minus M1 | 0.002 | -0.103 | 0.111 | 1000 |
| Calibration_slope | M3 minus M1 | 0.513 | 0.175 | 0.816 | 1000 |
| AUC | M3 minus M2 | 0.067 | 0.018 | 0.109 | 1000 |
| Brier | M3 minus M2 | -0.025 | -0.044 | -0.001 | 1000 |
| Calibration_intercept | M3 minus M2 | -0.002 | -0.129 | 0.130 | 1000 |
| Calibration_slope | M3 minus M2 | 0.799 | 0.406 | 1.261 | 1000 |
| AUC | M4 minus M1 | 0.002 | -0.054 | 0.058 | 1000 |
| Brier | M4 minus M1 | 0.015 | -0.011 | 0.041 | 1000 |
| Calibration_intercept | M4 minus M1 | 0.003 | -0.145 | 0.144 | 1000 |
| Calibration_slope | M4 minus M1 | 0.655 | 0.293 | 1.035 | 1000 |
| AUC | M3 minus M4 | 0.071 | 0.044 | 0.094 | 1000 |
| Brier | M3 minus M4 | -0.034 | -0.044 | -0.021 | 1000 |
| Calibration_intercept | M3 minus M4 | -0.001 | -0.069 | 0.077 | 1000 |
| Calibration_slope | M3 minus M4 | -0.141 | -0.398 | 0.068 | 1000 |

**Supplementary Table S11. Decision consequences at selected threshold probabilities.**

The action was prioritization for expedited tissue diagnosis within the existing BI-RADS 4 biopsy pathway. These results do not support withholding tissue diagnosis.

| **Model** | **Threshold** | **Prioritized, n** | **Prioritized, %** | **Malignant prioritized, n** | **Benign prioritized, n** | **Malignant not prioritized, n** | **Sensitivity** | **Specificity** | **PPV** | **NPV** | **Corrected net benefit** | **NB 95% CI lower** | **NB 95% CI upper** | **Net reduction per 100** |
| --- | --- | --- | --- | --- | --- | --- | --- | --- | --- | --- | --- | --- | --- | --- |
| M1 | 0.100 | 441 | 100.0 | 165 | 276 | 0 | 1.000 | 0.000 | 0.374 | - | 0.304 | 0.252 | 0.350 | -0.2 |
| M1 | 0.200 | 225 | 51.0 | 131 | 94 | 34 | 0.794 | 0.659 | 0.582 | 0.843 | 0.242 | 0.193 | 0.289 | 9.5 |
| M1 | 0.300 | 225 | 51.0 | 131 | 94 | 34 | 0.794 | 0.659 | 0.582 | 0.843 | 0.205 | 0.155 | 0.255 | 23.1 |
| M1 | 0.500 | 74 | 16.8 | 61 | 13 | 104 | 0.370 | 0.953 | 0.824 | 0.717 | 0.101 | 0.045 | 0.143 | 35.3 |
| M2 | 0.100 | 415 | 94.1 | 159 | 256 | 6 | 0.964 | 0.072 | 0.383 | 0.769 | 0.297 | 0.246 | 0.341 | -6.9 |
| M2 | 0.200 | 273 | 61.9 | 137 | 136 | 28 | 0.830 | 0.507 | 0.502 | 0.833 | 0.242 | 0.189 | 0.290 | 9.7 |
| M2 | 0.300 | 219 | 49.7 | 125 | 94 | 40 | 0.758 | 0.659 | 0.571 | 0.820 | 0.198 | 0.148 | 0.244 | 21.4 |
| M2 | 0.500 | 130 | 29.5 | 88 | 42 | 77 | 0.533 | 0.848 | 0.677 | 0.752 | 0.111 | 0.061 | 0.159 | 36.3 |
| M3 | 0.100 | 401 | 90.9 | 165 | 236 | 0 | 1.000 | 0.145 | 0.411 | 1.000 | 0.309 | 0.258 | 0.354 | 3.8 |
| M3 | 0.200 | 301 | 68.3 | 151 | 150 | 14 | 0.915 | 0.457 | 0.502 | 0.900 | 0.254 | 0.201 | 0.302 | 14.7 |
| M3 | 0.300 | 227 | 51.5 | 139 | 88 | 26 | 0.842 | 0.681 | 0.612 | 0.879 | 0.219 | 0.166 | 0.267 | 26.3 |
| M3 | 0.500 | 131 | 29.7 | 101 | 30 | 64 | 0.612 | 0.891 | 0.771 | 0.794 | 0.140 | 0.088 | 0.186 | 39.2 |
| M4 | 0.100 | 429 | 97.3 | 165 | 264 | 0 | 1.000 | 0.043 | 0.385 | 1.000 | 0.304 | 0.252 | 0.351 | -0.5 |
| M4 | 0.200 | 335 | 76.0 | 155 | 180 | 10 | 0.939 | 0.348 | 0.463 | 0.906 | 0.234 | 0.180 | 0.281 | 6.6 |
| M4 | 0.300 | 251 | 56.9 | 134 | 117 | 31 | 0.812 | 0.576 | 0.534 | 0.837 | 0.180 | 0.126 | 0.227 | 17.3 |
| M4 | 0.500 | 124 | 28.1 | 87 | 37 | 78 | 0.527 | 0.866 | 0.702 | 0.754 | 0.077 | 0.025 | 0.122 | 32.9 |

**Supplementary Table S12. Bootstrap selection stability of terms retained in the final lambda.1se model.**

| **Term** | **Full-data coefficient** | **Selection frequency** | **Bootstrap 2.5th percentile** | **Bootstrap 97.5th percentile** | **Bootstrap n** |
| --- | --- | --- | --- | --- | --- |
| Intercept | -3.443663 | 100.0% | -6.102 | -2.929 | 1000 |
| Architectural distortion: Yes | 0.075486 | 69.9% | 0.000 | 1.011 | 1000 |
| BI-RADS: BI-RADS 4B | 0.505122 | 98.3% | 0.069 | 1.259 | 1000 |
| BI-RADS: BI-RADS 4C | 1.776109 | 100.0% | 1.296 | 2.723 | 1000 |
| Normalized GLCM contrast | 0.995985 | 89.6% | 0.000 | 2.768 | 1000 |
| Circularity-deficit irregularity score | 2.345170 | 99.3% | 0.494 | 4.733 | 1000 |
| Contour serration score | 2.847925 | 99.9% | 1.282 | 4.783 | 1000 |

**Supplementary Table S13. Calcification morphology and distribution in lesions with calcification data.**

| **Descriptor** | **Level** | **Outcome** | **n** | **Percent** |
| --- | --- | --- | --- | --- |
| calcification_distribution | Diffuse | Benign | 15 | 14.0 |
| calcification_distribution | Diffuse | Malignant | 16 | 19.5 |
| calcification_distribution | Grouped | Benign | 31 | 29.0 |
| calcification_distribution | Grouped | Malignant | 23 | 28.0 |
| calcification_distribution | Linear | Benign | 14 | 13.1 |
| calcification_distribution | Linear | Malignant | 15 | 18.3 |
| calcification_distribution | Missing | Benign | 15 | 14.0 |
| calcification_distribution | Missing | Malignant | 10 | 12.2 |
| calcification_distribution | Regional | Benign | 23 | 21.5 |
| calcification_distribution | Regional | Malignant | 11 | 13.4 |
| calcification_distribution | Segmental | Benign | 9 | 8.4 |
| calcification_distribution | Segmental | Malignant | 7 | 8.5 |
| calcification_morphology | Amorphous | Benign | 45 | 42.1 |
| calcification_morphology | Amorphous | Malignant | 16 | 19.5 |
| calcification_morphology | Coarse heterogeneous | Benign | 19 | 17.8 |
| calcification_morphology | Coarse heterogeneous | Malignant | 9 | 11.0 |
| calcification_morphology | Fine linear/branching | Benign | 12 | 11.2 |
| calcification_morphology | Fine linear/branching | Malignant | 19 | 23.2 |
| calcification_morphology | Fine pleomorphic | Benign | 19 | 17.8 |
| calcification_morphology | Fine pleomorphic | Malignant | 22 | 26.8 |
| calcification_morphology | Missing | Benign | 12 | 11.2 |
| calcification_morphology | Missing | Malignant | 16 | 19.5 |

**Supplementary model equation**

**Model specification:** Full mammography-based LASSO logistic regression (alpha = 1; lambda.1se selected).

**Penalty parameters:** lambda.1se = 0.0437985910336; lambda.min = 0.0250631640856.

**Linear predictor:** LP = -3.4436631237 + 0.5051221864(BI-RADS 4B) + 1.7761088138(BI-RADS 4C) + 0.0754857523(architectural distortion: yes) + 2.3451697118(circularity-deficit irregularity score) + 2.8479250514(contour serration score) + 0.9959849598(normalized GLCM contrast).

**Predicted probability:** exp(LP) / [1 + exp(LP)].

**Coding and scaling:** Categorical predictors use treatment coding with the reference levels in Supplementary Table S5. Continuous predictors are entered in their original recorded units. The glmnet coefficients are reported on the original predictor scale despite internal standardization.

**Code and source data:** The analytic R code, aggregated model-output tables, and figure source data are supplied in a separate supplementary archive; patient-level data are not included.

**2. Supplementary Figures**

**Supplementary Figure S1. Predicted malignancy probabilities within BI-RADS subcategories.**

Distribution of bootstrap out-of-bag predicted malignancy probabilities from the full mammography-based LASSO model within BI-RADS 4A, 4B, and 4C, stratified by observed outcome. The BI-RADS 4C benign group was small (n = 13), and density shapes should be interpreted accordingly.
